# Serum neutrophil gelatinase-associated lipocalin levels predict the neurological outcomes of out-of-hospital cardiac arrest victims

**DOI:** 10.1186/s12872-017-0545-y

**Published:** 2017-05-08

**Authors:** Tadashi Kaneko, Motoki Fujita, Yasuaki Ogino, Takahiro Yamamoto, Ryosuke Tsuruta, Shunji Kasaoka

**Affiliations:** 1 Emergency and General Medicine, Kumamoto University Hospital, 1-1-1 Honjo, Chuo-ku, Kumamoto, 860-8556 Japan; 2 Advanced Medical Emergency and Critical Care Center, Yamaguchi University Hospital, Ube, Japan

**Keywords:** Neuron-specific enolase, Cerebral performance category, Post cardiac arrest syndrome

## Abstract

**Background:**

Serum neutrophil gelatinase-associated lipocalin (NGAL) is a well-known biomarker of acute kidney injury. Serum NGAL was recently proposed as a potential predictor of mortality in post cardiac arrest syndrome (PCAS) patients following out-of-hospital cardiac arrest (OHCA). However, the potential predictive value of NGAL for neurological outcomes is unknown. Therefore, we assessed the potential predictive value of NGAL for neurological outcomes after OHCA. We also compared its predictive value with that of neuron-specific enolase (NSE) as an established biomarker.

**Methods:**

Blood samples were prospectively collected from 43 PCAS patients following OHCA. Serum NGAL was measured on days 1 and 2, and NSE was measured on day 2. These biomarkers were compared between patients with favourable (cerebral performance category [CPC] 1–2) and unfavourable (CPC 3–5) outcomes. Receiver operating characteristic (ROC) curve analysis was performed.

**Results:**

Serum NGAL and NSE on day 2 (both *P* < 0.001), but not NGAL on day 1 (*P* = 0.609), were significantly different between the favourable and unfavourable groups. In ROC curve analysis, the sensitivity and specificity were 83% and 85%, respectively, for NGAL (day 2) at a cutoff value of 204 ng/mL and were 84% and 100% for NSE (day 2) at a cutoff value of 28.8 ng/mL. The area under the ROC curve of NGAL (day 2) was equivalent to that of NSE (day 2) (0.830 vs. 0.918). Additionally, the area under the ROC curve in subgroup of estimated glomerular filtration rate (eGFR) > 20 mL/min/1.73 m^2^ (*n* = 38, 0.978 vs. 0.923) showed the potential of NGAL predictability.

**Conclusions:**

Serum NGAL might predict the neurological outcomes of PCAS patients, and its predictive value was equivalent to that of NSE.

## Background

Between 2005 and 2012, more than 900,000 cases of out-of-hospital cardiac arrest (OHCA) occurred in Japan, but only about 1% of OHCA patients had favourable outcomes [1]. Therefore, assessing the prognosis of OHCA patients is important in order to identify and treat patients likely to have favourable outcomes, because most OHCA patients have an unfavourable outcome. Several chemical biomarkers have been examined in terms of the ability to predict the neurological outcomes, and neuron-specific enolase (NSE) and S100B protein are frequently measured for this purpose [2]. NSE is perhaps the most widely measured marker and several prognostic cutoff values have been proposed [3].

Neutrophil gelatinase-associated lipocalin (NGAL) is a member of the lipocalin superfamily that is expressed by neutrophils and epithelial cells. NGAL is an established biomarker of acute kidney injury (AKI) and several reviews have shown that NGAL has potential predictive value for AKI in septic patients [4, 5]. NGAL was also reported as a potential predictive biomarker of mortality and multiple organ dysfunction syndrome in septic patients [6].

Patients with return of spontaneous circulation (ROSC) after cardiac arrest (which includes OHCA) are treated like those with post cardiac arrest syndrome (PCAS) [7]. PCAS is sometimes referred to as sepsis-like syndrome. Several studies have revealed that blood NGAL concentrations are increased in PCAS patients, and that NGAL might predict the clinical outcomes of these patients, as already reported in sepsis patients [8, 9]. However, these studies were not sufficient to conclude that NGAL is a predictor of neurological outcomes. Then, it is also known that serum NGAL is affected by renal function of each patients, therefore, serum NGAL should be analysed with additional regulation by each patients’ kidney data. In the present study, we measured serum NGAL to assess whether it predicted the neurological outcomes of PCAS patients with additional regulation by kidney data. We also compared its predictive value against that of NSE measured at the recommended time as an established biomarker. This is the first report of presenting comparison of serum NGAL and NSE for neurological outcome with regulation by kidney function in PCAS case of OHCA.

## Methods

### Subjects

**Fig. 1:**
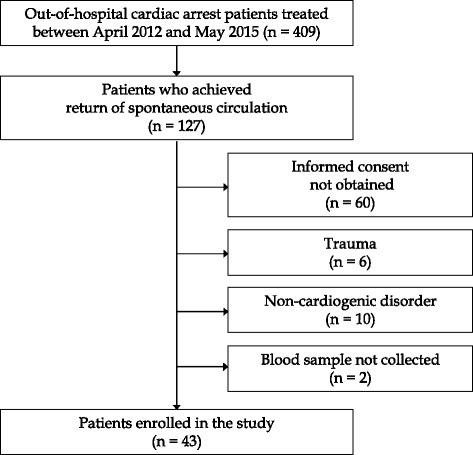
Patient disposition

The protocol of this clinical study was approved by the Institutional Review Boards of Kumamoto and Yamaguchi University Hospitals. OHCA patients who were admitted to the Emergency Medical Center at Yamaguchi University Hospital between April 2012 and May 2015 were included as the study group, and the disposition of patients is shown in Fig. 1. Among 409 patients with OHCA, ROSC was achieved in 127. Of these, 43 satisfied the eligibility criteria and informed consent for participation was obtained from the next of kin (Fig. 1).

Inclusion in this study did not alter the management of patients. Basically, all patients received ventilator management and blood pressure regulation (systolic blood pressure > 90 mmHg and mean arterial pressure > 70 mmHg). If patients received therapeutic hypothermia, they were sedated (midazolam 0.1–0.2 mg·kg^−1^·h^−1^ and fentanyl 1 μg·kg^−1^·h^−1^), administered a muscle relaxant (vecronium 0.05 mg·kg^−1^·h^−1^ or rocuronium 0.42 mg·kg^−1^·h^−1^) for ≥48 h, core body temperature was measured with a bladder probe, and therapeutic hypothermia was initiated using an intravascular cooling device (Thermogard; Alsius Inc., Irvine, CA, USA). If required, veno–arterial extracorporeal membrane oxygenation support was started before ROSC and was continued until spontaneous circulation had recovered sufficiently.

Neurological outcomes were evaluated as the Glasgow–Pittsburgh Cerebral Performance Category (CPC) at hospital discharge. The CPC comprises five categories: CPC1 (good recovery), CPC2 (moderate disability), CPC3 (severe disability), CPC4 (vegetative state), and CPC5 (death) [10]. In the present study, patients were divided into two groups based on the CPC at discharge as patients with favourable outcomes (CPC1–2, *n* = 20) and patients with unfavourable outcomes (CPC3–5, *n* = 23). The serum NGAL and NSE concentrations were compared between the two groups. In the present study, CPC was assessed by physician who was not relate to the patients’ treatment.

### Sample collection

This observational study involved prospective blood sample collection. Blood samples were collected from patients after ROSC via an arterial line on days 1 and 2. Day 1 was defined as the day after ROSC. Blood samples were centrifuged at 3000 rpm for 10 min at 4 °C, and then stored at −80 °C until further use.

### Assays

Serum NGAL levels were analysed twice in each sample using an enzyme-linked immunosorbent assay (ELISA; BioPorto, Hellerup, Denmark). Serum NSE levels were also analysed using an ELISA (Alpha Diagnostic International, San Antonio, TX, USA). NGAL was measured in samples obtained on days 1 and 2, and NSE was measured in samples obtained on day 2.

### Statistical analysis

All statistical analyses were performed using SPSS software version 23.0 (IBM, Armonk, NY, USA). Univariate analyses was performed with the Mann–Whitney *U* test for continuous variables and Fisher’s exact test for categorical variables. Receiver operating characteristic (ROC) curve analysis was used to evaluate the cutoff value for predicting unfavourable outcomes. The cutoff value was expressed as the left-uppermost point of the ROC curve, and the area under the ROC curve was measured to determine the predictive potential. The threshold of significance was set at *P* < 0.05.

In addition, the ROC curve analysis was also measured in subgroup of estimated glomerular filtration rate (eGFR) > 20 mL/min/1.73 m^2^. The subgroup (eGFR > 20 mL/min/1.73 m^2^) was thought as the group of having the potential kidney function to avoid dialysis.

## Results

The characteristics of the 43 patients are shown in Table 1. The favourable outcome group (i.e. CPC1–2) was younger (*P* = 0.011), included more patients with ventricular fibrillation or tachycardia rhythm (*P* = 0.023), and included more patients who received therapeutic hypothermia (*P* = 0.023), compared with the unfavourable outcome group (i.e. CPC3–5).

**Table 1:** Patient characteristics

Variables	CPC1–2 (*n* = 20)	CPC3–5 (*n* = 23)	*P* value
Age (years)	62 (52–66)	71 (57–79)	0.011
Male (%)	17 (85%)	16 (70%)	0.294
Witness (%)	16 (80%)	16 (70%)	0.501
Bystander CPR (%)	10 (50%)	7 (30%)	0.225
VF/VT (%)	17 (85%)	11 (48%)	0.023
Time from collapse to ROSC (min)	6 (5–10)	7 (0–15)	0.596
Therapeutic hypothermia	17 (85%)	11 (48%)	0.023
VA-ECMO assist	1 (5%)	5 (22%)	0.192
Creatinine (mg/dL)	0.94 (0.75–1.25)	1.20 (0.84–1.83)	0.301
eGFR (mL/min/1.73 m^2^)	61 (47–73)	48 (29–64)	0.144
CPC1	19	0	
CPC2	1	0	
CPC3	0	4	
CPC4	0	6	
CPC5	0	13	

Values are presented as the median (interquartile range) or n (%)

*CPC* cerebral performance category, *CPR* cardiopulmonary resuscitation, *VF* ventricular fibrillation, *VT* ventricular tachycardia, *ROSC* return of spontaneous circulation, *VA-ECMO* veno–arterial extracorporeal organ oxygenation, *eGFR* estimated glomerular filtration rate

**Fig. 2:**
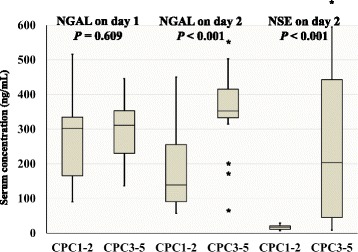
Box-plots of serum NGAL (days 1 and 2) and NSE (day 2) concentrations in the favourable outcome (CPC1–2) and unfavourable outcome (CPC3–5) groups. Serum NGAL on day 2 and NSE on day 2 were significantly different between the two groups (both *P* < 0.001). Asterisks denote outliers. CPC, cerebral performance category; NGAL, neutrophil gelatinase-associated lipocalin; NSE, neuron-specific enolase

Figure 2 shows the serum NGAL and NSE concentrations in the favourable and unfavourable outcome groups. There were significant differences between the favourable and unfavourable outcome groups in terms of serum NGAL on day 2 (favourable vs. unfavourable: 139 ng/mL vs. 352 ng/dL, *P* < 0.001) and NSE on day 2 (17 ng/mL vs. 204 ng/dL, *P* < 0.001).

**Fig. 3:**
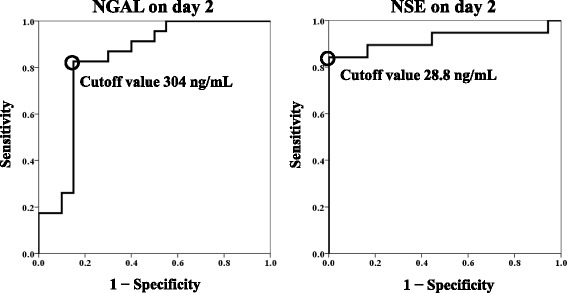
Receiver operating characteristic curves for serum NGAL and NSE for predicting unfavourable outcomes. The cutoff value was taken as the left-uppermost point. NGAL, neutrophil gelatinase-associated lipocalin; NSE, neuron-specific enolase

Figure 3 and Table 2 show the results of the ROC curve analysis of serum NGAL and NSE on day 2 for predicting unfavourable outcomes. The areas under ROC curves for NGAL (0.830, *P* < 0.001) and NSE (0.918, *P* < 0001) were statistically significant. Based on the left-uppermost points, the cutoff values were 304 ng/dL for NGAL on day 2 (sensitivity 83% and specificity 85%) and 28.8 ng/dL for NSE on day 2 (sensitivity 84% and specificity 100%).

**Table 2:** Results of receiver operating characteristic curve analysis

Variables	NGAL on day 2	NSE on day 2
AUC	0.830	0.918
95% CI	0.697–0.964	0.812–1.000
*P* value	<0.001	<0.001
Cutoff value (ng/mL)	304	28.8
Sensitivity	83%	84%
Specificity	85%	100%

*NGAL* neutrophil gelatinase-associated lipocalin, *NSE* neuron-specific enolase, *AUC* area under the curve, *CI* confidence interval

**Fig. 4:**
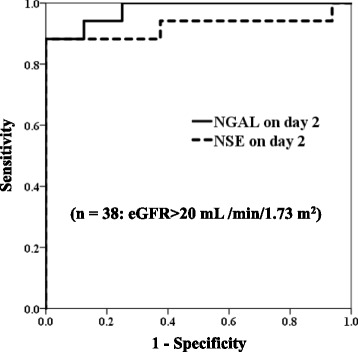
Receiver operating characteristic curves for serum NGAL and NSE for predicting unfavourable outcomes in the subgroup of eGFR > 20 mL/min/1.73 m^2^. NGAL, neutrophil gelatinase-associated lipocalin; NSE, neuron-specific enolase

Figure 4 and Table 3 show the results of the ROC curve analysis of serum NGAL and NSE on day 2 for predicting unfavourable outcomes in the subgroup of estimated glomerular filtration rate (eGFR) > 20 mL/min/1.73 m^2^. (*n* = 38) The areas under ROC curves for NGAL (0.978, *P* < 0.001) and NSE (0.923, *P* < 0001) were statistically significant. Based on the left-uppermost points, the cutoff values were 304 ng/dL for NGAL on day 2 (sensitivity 88% and specificity 100%) and 28.8 ng/dL for NSE on day 2 (sensitivity 88% and specificity 100%).

**Table 3:** Results of receiver operating characteristic curve analysis of patients with eGFR > 20 (mL/min/1.73 m^2^) (*n* = 38)

Variables	NGAL on day 2	NSE on day 2
AUC	0.978	0.923
95% CI	0.939–1.000	0.810–1.000
*P* value	<0.001	<0.001
Cutoff value (ng/mL)	304	28.8
Sensitivity	88%	88%
Specificity	100%	100%

*eGFR* estimated glomerular filtration rate, *NGAL* neutrophil gelatinase-associated lipocalin, *NSE* neuron-specific enolase, *AUC* area under the curve, *CI* confidence interval

## Discussion

Serum NGAL is a potential biomarker for the clinical outcomes of PCAS patients. As described below, serum NGAL predicted mortality in OHCA, and had potential predictive value for the neurological outcomes in some studies. However, because the studies did not compare its predictive value with that of other biomarkers, it was not possible to conclude that serum NGAL can predict the neurological outcomes of OHCA patients. Elmer et al. reported that serum NGAL was a stronger predictor of hospital mortality in cardiac arrest patients than other markers, such as NSE, S100B protein, and high-mobility group protein 1 [9]. Park et al. reported that serum NGAL predicted AKI in OHCA patients admitted to an intensive care unit, as well as the 30-day survival and neurological outcomes in these patients [8]. In the present study, serum NGAL on day 2 showed potential predictive value for the neurological outcomes at discharge of OHCA patients. However, serum NGAL on day 1 was not predictive of these outcomes, a result that differs from those reported by Park et al., who measured NGAL upon admission to the intensive care unit. In our study, 65% (28/43) of patients received therapeutic hypothermia, which might affect the NGAL concentration, as has been reported for other biomarkers [11, 12].

NSE is one of the most frequently measured biomarkers in PCAS patients. A recent prospective study recommended a cutoff value of 33 ng/mL for NSE measured between 24 and 72 h after ROSC in patients who did not undergo therapeutic hypothermia [13]. Additionally, a post hoc analysis of a recent randomized controlled study of therapeutic hypothermia showed that there was no NSE difference between the target temperature groups, but the cutoff value was >33 ng/mL [3]. In this study, we measured serum NSE on day 2, which was the recommended time in a previous study [3]. In our study, the cutoff value for NSE on day 2 (about 24–72 h) was 28.8 ng/mL, and the false-positive rate was 0%, below, which was lower than that in a previous study [3], possibly because most of the patients with CPC1–2 showed good recovery (CPC1) and the NSE concentration was not increased in the favourable outcome group.

In the present study, serum NGAL showed potential value for predicting the prognosis of PCAS patients, and it was significantly different between the favourable and unfavourable outcomes groups on day 2. However, based on the ROC curve analysis, serum NGAL on day 2 did not show sufficient sensitivity and specificity. At a cutoff value of 304 ng/mL, the false-positive rate was 24%. Moreover, the area under the ROC curve for NGAL (0.830) was equivalent to that of NSE (0.918). Therefore, our data did not show superiority of serum NGAL over serum NSE as a biomarker for predicting the neurological outcomes of PCAS patients.

Serum NGAL is well known as a biomarker for AKI, and it is possible that AKI might affect serum NGAL concentrations [4, 5]. Therefore, the predictive value of serum NGAL in PCAS patients with AKI is unclear. Our study included five patients whose estimated glomerular filtration rate was <20 mL/min/1.73 m^2^. Therefore, these five patients were eliminated from our data, the area under the ROC curve was 0.978 for serum NGAL, which was superior to that of NSE (0.923). At the cutoff value of 304 ng/mL for serum NGAL, the sensitivity and specificity were 88% and 100%, respectively, which were equivalent to those of NSE at a cutoff value of 28.8 ng/dL. Based on these data, if the effect of AKI can be eliminated, serum NGAL could be a useful predictive biomarker for PCAS patients.

Finally, further study is needed to solve these questions and investigate the potential of serum NGAL for predicting the prognosis of PCAS patients.

Our study has some limitations to mention. First, this study was performed at a single centre and involved a small number of patients, which may limit the strength of the conclusions. Second, the neurological outcomes were assessed at hospital discharge, and some patients experience neurological improvement after discharge. Third, the blood samples could be collected at any time of day, and could range by up to 24 h among patients.

## Conclusions

Serum NGAL could be potential biomarker for predicting the neurological outcomes of PCAS patients. The predictive value of serum NGAL was equivalent to serum NSE.

## Abbreviations

AKI: Acute kidney injury
CPC: Cerebral performance category
eGFR: Estimated glomerular filtration rate
ELISA: Enzyme-linked immunosorbent assay
NGAL: Neutrophil gelatinase-associated lipocalin
NSE: Neuron-specific enolase
OHCA: Out-of-hospital cardiac arrest
PCAS: Post cardiac arrest syndrome
ROC: Receiver operating characteristic
ROSC: Return of spontaneous circulation

## References

[1] Nagao K, Nonogi H, Yonemoto N (2016). Duration of prehospital resuscitation efforts after out-of-hospital cardiac arrest. Circulation.

[2] Rossetti AO, Rabinstein AA, Oddo M (2016). Neurological prognostication of outcome in patients in coma after cardiac arrest. Lancet Neurol.

[3] Stammet P, Collignon O, Hassager C (2015). Neuron-specific enolase as a predictor of death or poor neurological outcome after out-of-hospital cardiac arrest and target temperature management at 33°C and 36°C. J Am Coll Cardiol.

[4] Kim S, Kim HJ, Ahn HS (2016). Is plasma neutrophil gelatinase-associated lipocalin a predictive biomarker for acute kidney injury in sepsis patients? A systematic review and meta-analysis. J Cit Care.

[5] Zhang A, Cai Y, Wang PF (2016). Diagnosis and prognosis of neutrophil gelatinase-associated lipocalin for acute kidney injury with sepsis: a systematic review and meta-analysis. Crit Care.

[6] Wang B, Chen G, Zhang J (2015). Increased NGAL is associated with mortality and multiple organ dysfunction syndrome in severe sepsis and aseptic shock. Shock.

[7] Stub D, Bernard S, Duffy SJ (2011). Post cardiac arrest syndrome: a review of therapeutic strategies. Circulation.

[8] Park SO, Ahn JY, Lee YH (2016). Plasma neutrophil gelatinase-associated lipocalin as an early predicting biomarker of acute kidney injury and clinical outcomes after recovery of spontaneous circulation in out-of-hospital cardiac arrest patients. Resuscitation.

[9] Elmer J, Jeong K, Abebe KZ (2016). Serum neutrophil gelatinase-associated lipocalin predicts survival after resuscitation from cardiac arrest. Crit Care Med.

[10] Jennett B, Bond M (1975). Assessment of outcome after severe brain damage. Lancet.

[11] Sandroni C, Cavallaro F, Callaway CW (2013). Predictors of poor neurological outcome in adult comatose survivors of cardiac arrest: a systematic review and meta-analysis. Part 1: patients not treated with therapeutic hypothermia. Resuscitation.

[12] Sandroni C, Cavallaro F, Callaway CW (2013). Predictors of poor neurological outcome in adult comatose survivors of cardiac arrest: a systematic review and meta-analysis. Part 2: patients treated with therapeutic hypothermia. Resuscitation.

[13] Zandbergen EG, Hijdra A, Koelman JH (2006). Prediction of poor outcome within the first 3 days of postanoxic coma. Neurology.

